# Supplementary material for: A resource of variant effect predictions of single nucleotide variants in model organisms
Source: Mol Syst Biol. 2018 Dec 20;14(12):e8430. doi: 10.15252/msb.20188430 (PMC6301329; doi:10.15252/msb.20188430)
Supplement: Supplementary file 1 — Expanded View Figures PDF [file MSB-14-e8430-s001.pdf]

Expanded View Figures

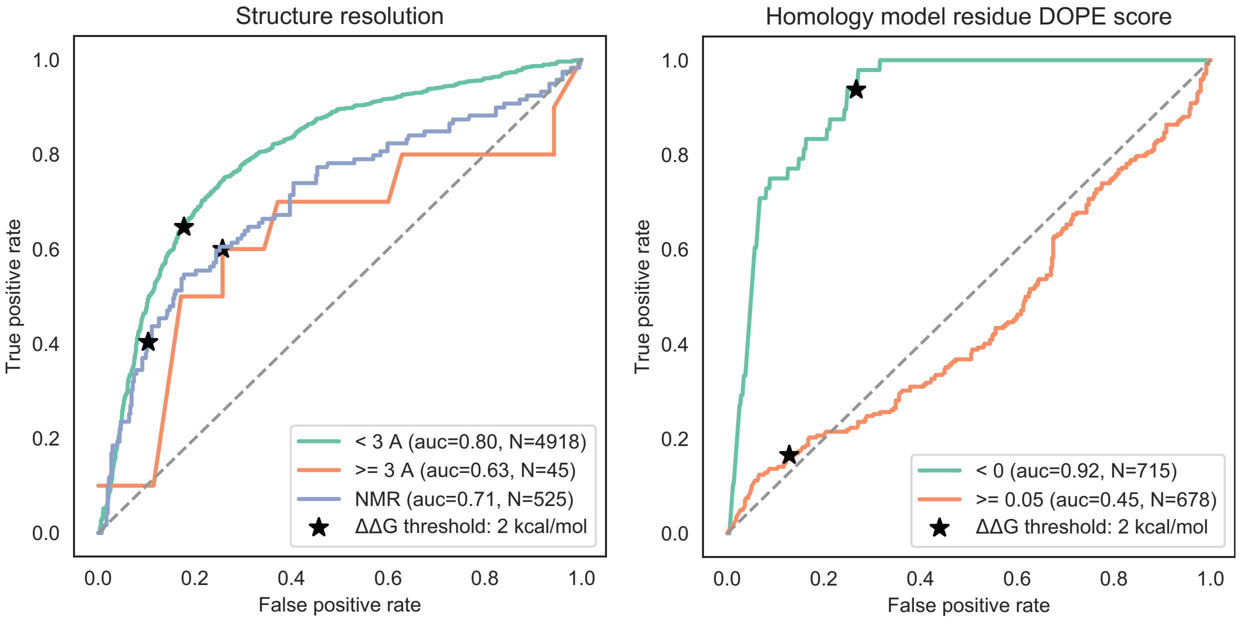

Figure EV1. Impact of structural models on structure-based variant effect prediction.

We obtained from the ProTherm database variants with experimentally determined impact on stability and classified them as destabilizing if $\Delta\Delta G > 2$ and not destabilizing otherwise. FoldX-based predictions were tested on their capacity to discriminate between these two classes of variants using different types of experimental models and regions within homology models with different predicted quality.

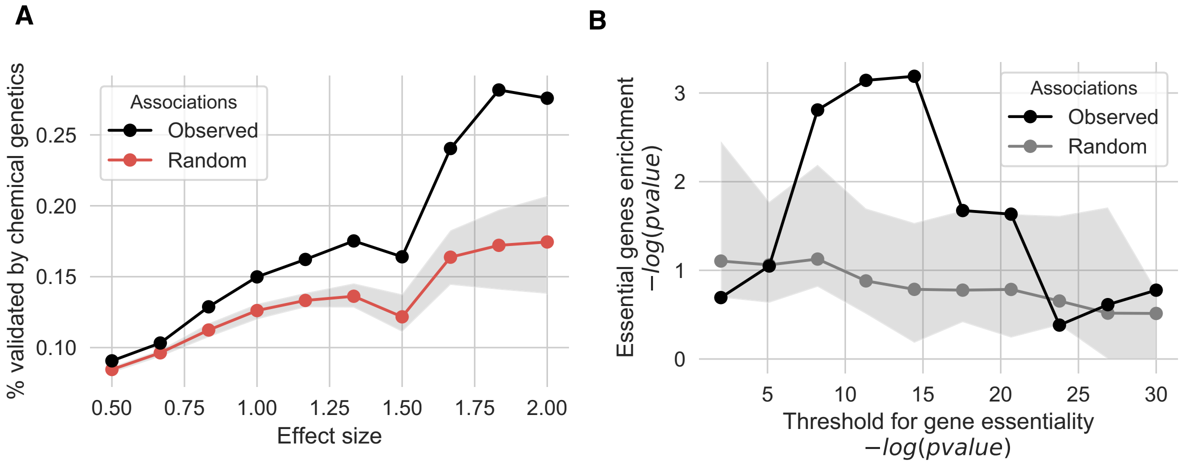

Figure EV2. Gene and protein complex-level phenotype association analysis show significant but modest enrichment in prior knowledge of gene KO growth phenotypes.

A The fraction of gene–phenotype associations that is validated by chemical genetic information derived from gene-deletion experiments. Shaded area reports the interquartile range over 100 iterations. The significance of the observed overlap was tested using permutation testing.

B Associations between protein complexes and conditions were benchmarked by calculating the enrichment of previously known gene-condition associations from gene-deletion studies. Shaded area reports the interquartile range over 100 iterations. An enrichment was observed for some cut-offs for the gene-deletion condition-dependent essentiality but only found to be better than random expectation for stringent cut-off.

© 2018 The Authors
